# Disseminated CNS tuberculosis in SLE without anti-TNF exposure: implications for LTBI screening in endemic settings—case report

**DOI:** 10.1016/j.ero.2026.100264

**Published:** 2026-10-26

**Authors:** Effie Kamadi, Mark Hawken, Reena Shah, Fredrick Otieno

**Affiliations:** Department of Medicine, Aga Khan University Medical College of East Africa, Nairobi, Kenya

## Abstract

Patients with systemic lupus erythematosus (SLE) are vulnerable to tuberculosis (TB) because of disease-related immune dysfunction, immunosuppressive therapy, and background exposure in TB-endemic settings. Although latent TB infection (LTBI) screening is routinely recommended before biologic and targeted synthetic disease-modifying antirheumatic drugs (DMARDs), guidance for glucocorticoids, conventional DMARDs, and other immunosuppressive therapies is less prescriptive. We report 2 HIV-negative Kenyan women with established SLE who developed disseminated extrapulmonary TB after approximately 12 and 20 months of mycophenolate mofetil therapy, respectively, while also receiving maintenance prednisolone 10 mg daily. The second patient had also received rituximab 7 months before TB diagnosis. In case 1, cerebrospinal fluid (CSF) GeneXpert *Mycobacterium tuberculosis* (MTB)/rifampicin and gluteal-abscess culture-confirmed disseminated central nervous system (CNS) TB with bilateral gluteal involvement. In case 2, lymph-node histology and culture-confirmed TB in the setting of widespread lymphadenopathy and splenic microabscesses; despite negative CSF investigations, neuroimaging demonstrated a cerebral lesion radiologically consistent with tuberculoma. Both patients received anti-TB therapy with adjunctive corticosteroids for CNS disease. Both patients demonstrated eventual clinical and radiological improvement. These cases demonstrate that severe disseminated TB, including CNS involvement, may occur in patients with SLE receiving nontumour necrosis factor (TNF) immunosuppressive regimens in TB-endemic settings. Although causal effects of individual immunosuppressive agents cannot be established, the cases support structured TB-risk assessment and a low threshold for LTBI testing before prolonged corticosteroid exposure or combination immunosuppression. Risk-stratified national guidance specifying therapy-related triggers for LTBI assessment may improve implementation and reduce preventable TB morbidity in high-burden settings.

## BACKGROUND

Tuberculosis (TB) remains a major public-health concern within the World Health Organization (WHO) African Region. In 2024, an estimated 2.62 million cases of TB were reported in the region. Kenya is 1 of WHO’s 30 high TB-burden countries with an estimated TB incidence of 207 per 100,000 population [1]. Patients with systemic lupus erythematosus (SLE) are especially susceptible to TB. A systematic review and meta-analysis of 27 studies, including 17 from 5 WHO high TB-burden countries, reported a pooled prevalence of TB disease of 4% among patients with SLE. Prevalence was notably higher in high TB-burden settings (5% vs 2%), with extrapulmonary and disseminated TB each occurring in approximately 1% of patients [2]. TB incidence among patients with SLE has been higher in African studies and in high TB-burden countries [3,4]. This propensity likely reflects a combined interplay of SLE-associated immune dysfunction, exposure to immunosuppressive therapy, and a high background risk of TB infection in endemic settings [5,6].

The 2022 European Alliance of Associations for Rheumatology (EULAR) recommendations for screening and prophylaxis of chronic and opportunistic infections strongly recommend latent TB infection (LTBI) screening before biologic and targeted synthetic disease-modifying antirheumatic drugs (DMARDs). In addition, they suggest that screening should be considered before conventional DMARDs, immunosuppressants, and glucocorticoids with no defined dose or duration threshold [7]. The Kenyan Guidelines on Programmatic Management of Tuberculosis Preventive Therapy similarly identify patients receiving immunosuppressive therapy as an at-risk population without specifying therapy-related thresholds [8]. This uncertainty is particularly consequential in TB-endemic settings, where the burden of TB in SLE is substantial. In a South African cohort of 512 patients with SLE, TB occurred in 14.1% of patients, with extrapulmonary disease accounting for 58.3% of episodes [9].

Against this background, we report 2 cases of microbiologically confirmed disseminated TB with central nervous system (CNS) involvement in patients with established SLE receiving nontumour necrosis factor (TNF) immunosuppressive therapy in a TB-endemic setting (Table 1). These cases highlight the potential for severe disseminated TB in patients with SLE receiving cumulative non-TNF immunosuppression and underscore the need for careful, context-specific LTBI assessment.

**Table 1:** Baseline clinical characteristics and immunosuppressive exposures preceding tuberculosis diagnosis

Characteristic	Case 1	Case 2
Age (y), sex	44, F	58, F
Year of SLE diagnosis	2011	2021
Relevant SLE manifestations	None, stable disease	ITP
Complement levels at TB diagnosis	C3 1.61 g/L; C4 0.49 g/L	C3 1.89 g/L; C4 0.40 g/L
Anti-ds DNA antibody	Not available	Not available
MMF at TB diagnosis	1 g twice daily	1 g twice daily
Prednisolone at TB diagnosis	10 mg once daily	10 mg once daily
Cumulative glucocorticoid burden	Intra-articular methylprednisolone 80 mg, approximately 4 wk before admission	- Dexamethasone 8 mg twice daily for 5 d; IV methylprednisolone 1 g both administered approximately 7 mo before TB diagnosis - Prednisolone 30 mg daily for approximately 6 wk, followed by tapering to 10 mg once daily
Hydroxychloroquine at TB diagnosis	Temporarily discontinued	200 mg twice daily
Biologic DMARD exposure	None known	Two rituximab infusions administered 7 mo before TB diagnosis
HIV serology	Negative	Negative
Identified TB contact	None known	None known
Psychosocial history	Married, nonsmoker with no significant alcohol use; attained college-level education. There was no significant familial medical history	Married; social alcohol use reported; attained master’s degree; and familial history of Hypertension

DMARD, disease-modifying antirheumatic drug; ITP, immune thrombocytopenia; IV, intravenous; MMF, mycophenolate mofetil; SLE, systemic lupus erythematosus; TB, tuberculosis.

Laboratory reference ranges were 0.90-1.70 g/L for C3 and 0.12-0.36 g/L for C4. Complement levels were not reduced in either patient at TB diagnosis; anti-double-stranded DNA antibody testing was unavailable in both cases.

## CASE 1 PRESENTATION

A 44-year-old woman with established SLE, diagnosed in 2011, was clinically stable on mycophenolate mofetil (MMF) 1 g twice daily and prednisolone 10 mg daily. Four weeks before admission, she had received an intra-articular methylprednisolone injection (80 mg) for right trochanteric bursitis. Her comorbidities included hypertension and chronic kidney disease (CKD) stage G3b (estimated glomerular filtration rate 38 mL/min/1.7 3m^2^) secondary to obesity-related focal segmental glomerulosclerosis, with a baseline creatinine of approximately 150 µmol/L. She presented with a 5-day history of gastrointestinal symptoms, malaise, chills, and fever, shortly after returning from Northern Ethiopia. No TB contact was identified. Initial investigations showed neutrophilic leukocytosis, normocytic anaemia, and stable CKD. During admission, per vaginal bleeding prompted pelvic magnetic resonance imaging (MRI), which incidentally demonstrated bilateral gluteal collections radiologically consistent with abscesses. Persistent fever was followed by new-onset focal seizures of the left upper limb and diplopia, prompting urgent neuroimaging and cerebrospinal fluid (CSF) examination.

### Investigations

#### Imaging

Pelvic MRI, performed for investigation of per vaginal bleeding, incidentally demonstrated bilateral gluteal collections consistent with abscesses, as described above. Subsequent brain MRI demonstrated leptomeningeal enhancement with a right precentral ring-enhancing lesion and associated oedema, vasculopathic lacunar infarcts, and enhancement of the cauda equina nerve roots consistent with tuberculous radiculopathy (Fig 1).

**Figure 1:**
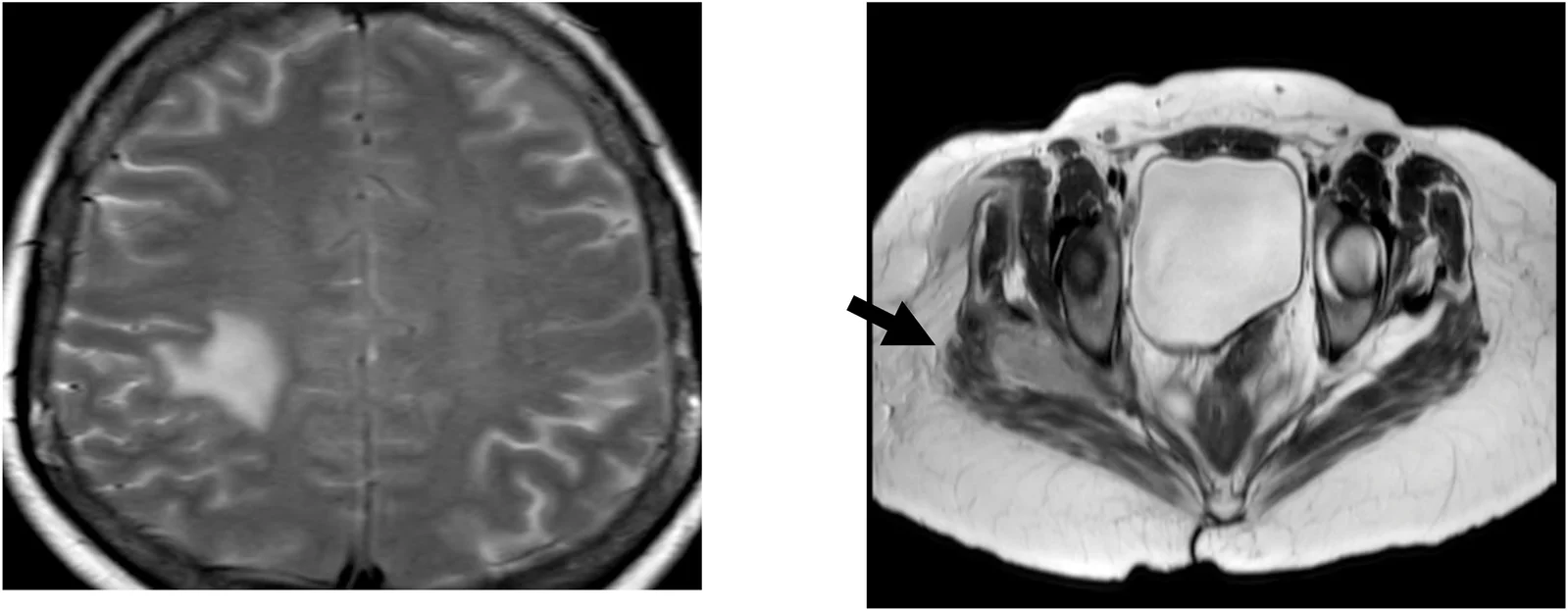
Left—brain MRI demonstrating the right precentral gyrus ring-enhancing tuberculoma with surrounding oedema and leptomeningeal enhancement. Right—pelvic MRI demonstrating a gluteal collection (arrow) consistent with an abscess. MRI, magnetic resonance imaging.

#### CSF analysis

CSF analysis showed a neutrophilic pleocytosis (100 cells/mm³), low glucose (1.50 mmol/L), and elevated lactate (5.16 mmol/L). CSF GeneXpert *M. tuberculosis*/rifampicin (GeneXpert MTB/RIF) was positive for *M. tuberculosis* complex, with no rifampicin resistance detected.

#### Microbiological confirmation

Image-guided drainage of the gluteal abscesses yielded acid-fast bacilli (AFB) smear-positive material (3+), with positive GeneXpert MTB/RIF and culture-confirmed *M. tuberculosis* complex. The isolate was susceptible to isoniazid, rifampicin, ethambutol, and streptomycin. She had no respiratory symptoms, and chest imaging showed no pulmonary involvement. Sputum was therefore not collected. A diagnosis of disseminated extrapulmonary TB was established, with CNS involvement manifesting as tuberculous meningitis, tuberculoma, and cerebrovascular infarcts, as well as cauda equina radiculopathy and bilateral gluteal abscesses. Key diagnostic features are summarised in Table 2.

**Table 2:** The diagnostic evidence and anatomical distribution of tuberculosis in both patients

Diagnostic domain	Case 1	Case 2
Neurological presentation	New focal seizures and diplopia	Right lower-limb UMN signs and focal seizures
CNS imaging	Leptomeningeal enhancement; right precentral gyrus tuberculoma; and vasculopathic lacunar infarcts	Left parafalcine ring-enhancing frontal lesion with surrounding oedema, radiologically consistent with tuberculoma
CSF findings	White-cell count 100 cells/mm³, neutrophil predominance; low glucose; elevated lactate	White-cell count < 5 cells/mm³; normal glucose; markedly elevated protein
CSF mycobacterial testing	GeneXpert MTB/RIF positive; rifampicin resistance not detected	AFB smear negative; GeneXpert MTB/RIF negative; mycobacterial culture negative
Extraneural TB sites	Cauda equina nerve roots; bilateral gluteal abscesses	Supraclavicular, axillary, retroperitoneal, and iliac lymphadenopathy; splenic microabscesses
Extraneural microbiological evidence	Gluteal aspirate AFB smear 3+; GeneXpert MTB/RIF positive; culture-confirmed *M. tuberculosis* complex	Inguinal lymph-node culture-confirmed *M. tuberculosis* complex
Histopathology	Not applicable	Necrotising granulomatous lymphadenitis
Drug-susceptibility testing	Susceptible to isoniazid, rifampicin, ethambutol, and streptomycin; pyrazinamide susceptibility unavailable	Pan-susceptible
Pulmonary involvement	No respiratory symptoms and no pulmonary lesions on chest imaging	No respiratory symptoms and no pulmonary lesions on chest imaging
Final diagnostic formulation	Culture-confirmed disseminated extrapulmonary TB with microbiologically confirmed CNS involvement	Culture-confirmed disseminated TB with a cerebral lesion radiologically consistent with tuberculoma

AFB, acid-fast bacilli; CNS, central nervous system; CSF, cerebrospinal fluid; MTB/RIF, *M. tuberculosis*/rifampicin; TB, tuberculosis; UMN, upper motor neuron

In case 2, CNS involvement was not microbiologically confirmed; the diagnosis of tuberculoma was based on compatible neuroimaging in the setting of culture-confirmed disseminated TB.

### Management

Antituberculous therapy (ATT) with rifampicin, isoniazid, pyrazinamide, and ethambutol was initiated shortly after admission, with pyridoxine supplementation. Dexamethasone was added for CNS disease, replacing maintenance prednisolone, whereas MMF was continued. There was no clinical evidence of an active SLE flare. The gluteal abscesses were drained, and anticonvulsant therapy was initiated for seizure control. The treatment course was complicated by syndrome of inappropriate antidiuretic hormone secretion–associated hyponatraemia, neuropathic symptoms, anticonvulsant intolerance, and bilateral femoral-head avascular necrosis identified during follow-up.

### Outcome and follow-up

She demonstrated progressive clinical and radiological improvement during ATT. Serial imaging showed reduction of the cerebral tuberculoma, resolution of leptomeningeal and cauda equina enhancement, and complete resolution of the gluteal collections. At last radiological follow-up, only a faint residual enhancing focus in the right precentral gyrus remained, interpreted as post-treatment change (Fig 2). Dexamethasone was gradually tapered and anticonvulsant therapy was discontinued following completion of ATT. She remained clinically stable on follow-up, with ongoing multidisciplinary care.

**Figure 2:**
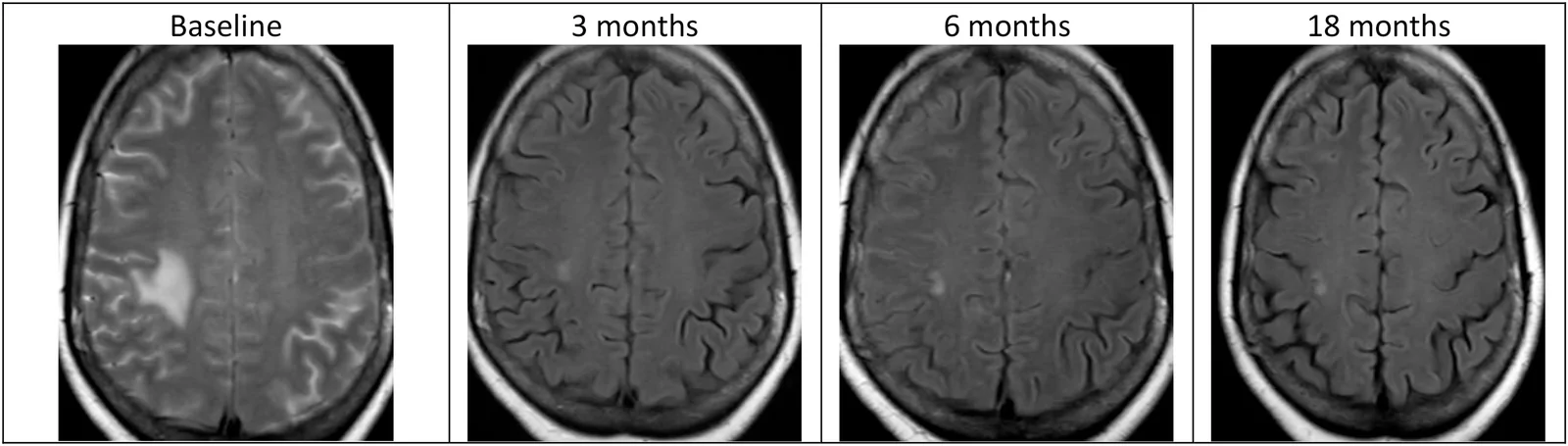
Serial brain MRI demonstrating progressive resolution of the right precentral gyrus tuberculoma from baseline to last follow-up. Leptomeningeal enhancement and associated oedema resolved with treatment, leaving a small residual post-treatment enhancing focus. MRI, magnetic resonance imaging.

## CASE 2 PRESENTATION

A 58-year-old woman with established SLE diagnosed in 2021 with comorbid erythema nodosum was on long-term hydroxychloroquine (HCQ) 200 mg twice daily and prednisolone 10 mg daily. She developed SLE-associated immune thrombocytopenia (ITP), which required treatment escalation with MMF 1 g twice daily introduced approximately 20 months before TB diagnosis, as well as high-dose oral corticosteroids, intravenous methylprednisolone, and rituximab administered approximately 7 months before TB diagnosis. At the time of TB diagnosis, she was receiving MMF 1 g twice daily, HCQ 200 mg twice daily, and prednisolone 10 mg daily. She presented with a 1-week history of right lower-limb weakness and intermittent focal jerks, associated with fatigue, 7 kg unintentional weight loss over 3 months, and fever. Examination demonstrated upper motor neuron signs in the right lower limb, prompting neurological evaluation.

### Investigations

#### Neuroimaging

Spinal MRI demonstrated retroperitoneal and bilateral iliac lymphadenopathy, with a normal spinal cord and cauda equina. Brain MRI showed a left parafalcine ring-enhancing lesion in the superior frontal lobe with surrounding oedema.

#### Cross-sectional imaging

Computed tomography of the chest, abdomen, and pelvis further demonstrated necrotic supraclavicular, axillary, retroperitoneal, and bilateral iliac lymphadenopathy, with splenic microabscesses, confirming widespread disseminated disease.

#### CSF analysis

CSF analysis showed markedly elevated protein at 1.61 g/L (reference range 0.15-0.45 g/L) with normal glucose and no pleocytosis; AFB smear, GeneXpert MTB/RIF, and mycobacterial culture were negative.

#### Microbiological and histological confirmation

Core biopsy of a right inguinal lymph node showed necrotising granulomatous lymphadenitis. Although GeneXpert MTB/RIF on lymph-node tissue was negative, mycobacterial culture-confirmed pan-susceptible *M. tuberculosis* complex. A diagnosis of culture-confirmed disseminated drug-susceptible TB was established, based on *M. tuberculosis* isolated from inguinal lymph-node tissue with necrotising granulomatous histology, together with a left frontal lesion radiologically consistent with tuberculoma. The patient had no respiratory symptoms and no pulmonary lesions on chest imaging; sputum samples were not collected. Key diagnostic features are summarised in Table 2.

### Management

Treatment with rifampicin, isoniazid, pyrazinamide, ethambutol, as well as pyridoxine and adjunctive dexamethasone for CNS disease was initiated shortly after admission. MMF and HCQ were continued for SLE control, and antiseizure therapy was commenced. Following enlargement of the cerebral lesion on subsequent MRI at 3 months (Fig 3), treatment was intensified after multidisciplinary review with high-dose rifampicin (total 1200 mg/d), levofloxacin 500 mg daily, and prednisolone 40 mg daily. Treatment was complicated by liver enzyme derangement, which contributed to a period of suboptimal rifampicin dosing, as well as recurrent focal seizures requiring adjustment of antiseizure therapy. Although brain biopsy was considered because of progressive lesion enlargement, it was deferred in view of clinical stability and partial extracranial radiological response.

**Figure 3:**
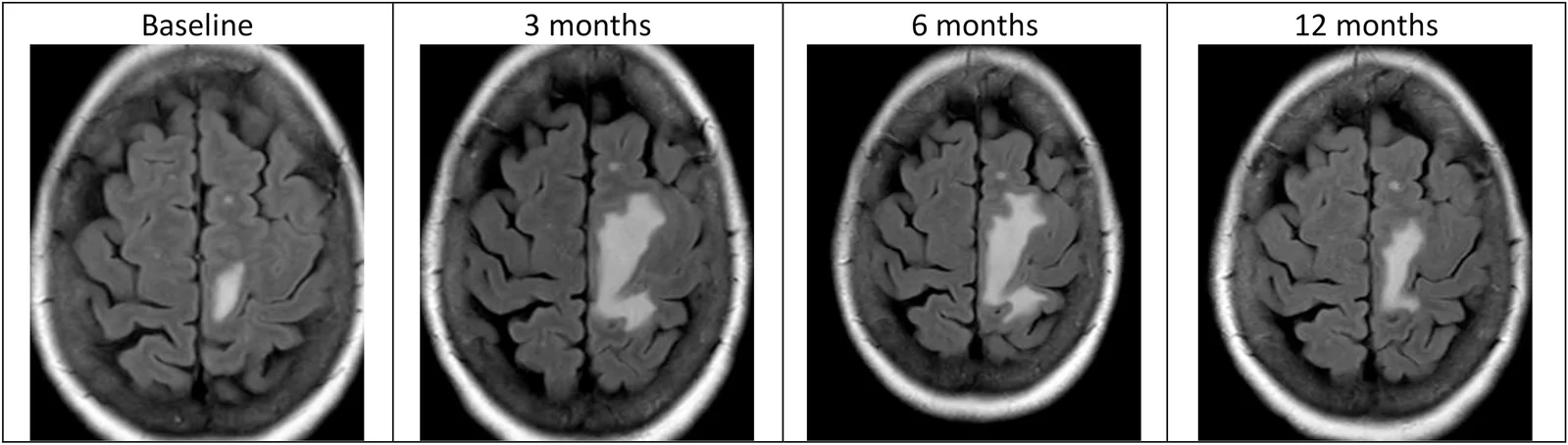
Serial brain MRI demonstrating initial enlargement and subsequent partial resolution of the left frontal tuberculoma during antituberculous therapy. MRI, magnetic resonance imaging.

### Outcome and follow-up

Following initiation of ATT and dexamethasone, she showed clinical improvement with partial radiological resolution of the lymphadenopathy and splenic microabscesses. The cerebral lesion initially enlarged despite treatment but subsequently decreased in size (from 3.8 to 2.7 mL) with reduced perilesional oedema at last radiological review (Fig 3). At her most recent clinical follow-up, she was afebrile, seizure-free, and functionally independent. Her SLE was clinically stable, and ITP was in remission. ATT was ongoing at the time of reporting, with multidisciplinary follow-up continuing.

## DISCUSSION

We report 2 cases of microbiologically confirmed extrapulmonary TB with disseminated disease and CNS involvement in patients with established SLE with no documented concurrent clinical SLE flare. In the first case, disease involved the CNS (tuberculoma, meningitis, and cerebrovascular infarction), cauda equina (radiculopathy), and bilateral gluteal region (abscesses), with *M. tuberculosis* identified in both CSF (GeneXpert) and gluteal-abscess material (positive culture). In the second case, lymph-node culture and histology confirmed *M. tuberculosis* infection in the setting of widespread lymphadenopathy, splenic microabscesses, and a cerebral lesion radiologically consistent with tuberculoma. Neither patient had received TNF inhibitor therapy. Case 2 had, however, received rituximab 7 months before TB diagnosis. These cases highlight the potential for severe disseminated TB in patients with SLE receiving non-TNF immunosuppressive regimens and underscore the importance of context-specific LTBI assessment in TB-endemic settings.

TB susceptibility in SLE is likely multifactorial, reflecting disease-associated immune dysregulation, exposure to immunosuppressive therapy, and background TB exposure in endemic settings [5]. Although the independent contribution of individual noncorticosteroid immunosuppressive agents remains uncertain, glucocorticoid exposure is the most consistently identified treatment-related risk factor. In a recent systematic review and meta-analysis, patients with SLE who developed TB had higher cumulative glucocorticoid exposure, whereas methylprednisolone pulse therapy was associated with increased odds of TB disease; associations with other immunosuppressive agents were inconclusive [2]. In the present cases, the relevant exposures differed: the first patient had long-term MMF and prednisolone 10 mg daily, with recent intra-articular methylprednisolone exposure, whereas the second had ongoing MMF and prednisolone with previous high-dose corticosteroid therapy and rituximab exposure.

TNF is central to granuloma maintenance and host control of *M.* tuberculosis. This accounts for the well-established TB reactivation risk associated with TNF inhibitors. In contrast, rituximab has not demonstrated the same marked TB reactivation signal, although evidence is largely derived from non-SLE populations and in lower TB-burden settings [10,11]. In case 2, the contribution of prior rituximab exposure should therefore be interpreted in the context of cumulative immunosuppression with high-dose steroids as well as ongoing MMF therapy in a TB-endemic setting. Evidence linking MMF to TB overall in SLE remains limited. In a South African retrospective cohort, MMF was not an independent predictor of TB when patients with SLE and TB were compared with SLE controls. However, among patients with SLE who developed TB, prior MMF exposure was independently associated with extrapulmonary rather than pulmonary disease. This finding may reflect confounding by disease severity and concurrent immunosuppression [9].

Historically, TB preventive strategies have been strongly emphasised before TNF inhibitor therapy. As noted, current EULAR recommendations leave screening before non-TNF immunosuppression dependent on individual TB-risk factors, local epidemiology, as well as treatment dose and duration. There are no defined thresholds for specific agents or combinations [7]. Both these cases demonstrate that severe disseminated TB may occur despite a prednisolone dose below the commonly cited threshold of >15 mg daily for >4 weeks [7] in the context of concurrent MMF therapy and residence in a TB-endemic setting. They support a low threshold for structured TB-risk assessment and consideration of LTBI testing before prolonged and/or combination non-TNF inhibitor immunosuppression in high-burden settings.

Notably, Kenyan TB preventive therapy guidelines similarly identify patients receiving immunosuppressive therapy as an at-risk population and recommend exclusion of active TB before preventive treatment; however, they do not define therapy-specific thresholds such as corticosteroid dose or duration, individual agents such as MMF or rituximab, or combination immunosuppressive regimens [8]. Accordingly, Kenyan recommendations leave an implementation gap where they do not provide a specific operational framework for determining when non-TNF immunosuppression should prompt LTBI assessment and, where indicated, TB preventive treatment.

Diagnosing CNS TB in immunosuppressed patients requires integration of CSF findings, neuroimaging, and evidence of TB at extraneural sites, as no individual negative CSF test excludes the diagnosis [12]. In case 2, culture confirmation from an inguinal lymph node was decisive despite negative CSF investigations, underscoring the importance of systematic extraneural sampling when CNS TB is suspected.

The enlargement of the cerebral lesion observed in case 2 during the first 3 months of ATT warrants specific consideration. Paradoxical reactions during TB therapy—defined as clinical or radiological worsening of pre-existing tuberculous lesions following appropriate treatment [13]—are a recognised phenomenon in CNS TB that are thought to reflect an exaggerated immune response. Among HIV-negative patients with CNS TB, the reported incidence of paradoxical reactions ranges from 12.7% to 64.7% [14]. In case 2, lesion enlargement occurred after initiation of antitubercular treatment but in the context of concurrent MMF therapy as well as suboptimal rifampicin dosing due to hepatotoxicity. However, in the absence of new extracranial sites of disease or clinical deterioration, a paradoxical reaction remains a leading diagnostic consideration. This is supported by the eventual reduction in lesion size following optimisation of ATT. Clinicians should, therefore, be aware that lesion enlargement during ATT does not necessarily indicate treatment failure and should be interpreted in the full clinical context [14,15].

These cases have several strengths, including microbiological confirmation of TB in both patients, detailed serial radiological follow-up, and prolonged clinical observation in a TB-endemic setting that is under-represented in existing literature. However, important limitations must be acknowledged. As a 2 case series, the findings are hypothesis-generating and cannot establish causality between specific immunosuppressive agents and TB reactivation. LTBI status was not documented before immunosuppression initiation in either patient. Sputum samples were not collected from either patient given the absence of respiratory symptoms and normal chest imaging, although this represents a diagnostic limitation given reported sputum positivity rates of approximately 13% in extrapulmonary TB [16]. In case 2, CNS involvement remains radiologically presumed rather than histologically confirmed. Patient perspectives were not obtainable for inclusion in this report.

## CONCLUSION

These cases illustrate that severe disseminated TB with CNS involvement may occur in patients with SLE receiving non-TNF immunosuppressive regimens in TB-endemic settings. A causal contribution of any individual immunosuppressive agent cannot be established; however, these cases support structured TB-risk assessment and a low threshold for LTBI testing before prolonged corticosteroid exposure or combination immunosuppression. Future updates to TB preventive therapy guidelines could consider a risk-stratified approach to LTBI assessment before immunosuppression, defining locally appropriate criteria for patients receiving glucocorticoids, conventional DMARDs, and other immunosuppressive agents. This would translate the current broad designation of immunosuppression as a risk category into a practical framework for identifying patients who should undergo LTBI testing and, where indicated, receive preventive treatment.

## Contributors

EK conceptualised the manuscript, collected clinical data, and drafted the manuscript. MH reviewed and edited the manuscript. RS reviewed and edited the manuscript. FO supervised the manuscript and provided clinical oversight for both cases. All authors read and approved the final manuscript.

## CRediT authorship contribution statement

**Effie Kamadi:** Writing – review & editing, Writing – original draft, Data curation, Conceptualization. **Mark Hawken:** Writing – review & editing, Supervision. **Reena Shah:** Writing – review & editing, Validation, Supervision. **Fredrick Otieno:** Writing – review & editing, Supervision, Project administration.

## Competing interests

All authors declare that they have no competing interests.
